# Hepatitis B Virus: Epidemiology, Prophylaxis, Therapy, Clinical Outcomes, and Novel Therapeutic Directions

**DOI:** 10.3390/biom16091290

**Published:** 2026-09-07

**Authors:** Uzair Iqbal, Khadija Khalid, Yunus Yukselten, Farooq Ahmad, Haseeb Ahmad, Abdur Rehman Khalid, Mohamed Shaltout, Richard E. Sutton

**Affiliations:** Section of Infectious Diseases, Department of Medicine, Yale School of Medicine, New Haven, CT 06520, USA; uzair.iqbal@yale.edu (U.I.); khadija.khalid@yale.edu (K.K.); yunus.yukselten@yale.edu (Y.Y.); farooq.ahmad@yale.edu (F.A.); haseeb.ahmad@yale.edu (H.A.); abdurrehman.khalid@yale.edu (A.R.K.); mohamed.shaltout@yale.edu (M.S.)

**Keywords:** hepatitis B virus (HBV), chronic hepatitis B, functional cure, cccDNA, novel therapeutic strategies

## Abstract

Hepatitis B virus (HBV) infection is a worldwide health concern that infects nearly 254 million people globally and causes more than 1 million deaths annually. The highest prevalence is seen in sub-Saharan Africa and the Western Pacific region. Cirrhosis, liver failure, and hepatocellular carcinoma (HCC) are reported as leading complications of chronic HBV. The route of transmission of this infection is mainly by exposure to infected blood and bodily fluids. Transmission from mother-to-child remains the predominant route in highly endemic areas. Vaccination has significantly reduced HBV seroprevalence and complications. However, incomplete vaccination of newborns continues to be a major obstacle to elimination of the disease. Current prevention strategies include universal vaccination, perinatal prophylaxis with hepatitis B immune globulin, and maternal antiviral therapy in pregnant women with high viral load. The management of chronic hepatitis B virus infection predominantly depends on nucleoside analogs, including entecavir, tenofovir disoproxil fumarate, and tenofovir alafenamide, as well as pegylated interferon alfa. These therapies effectively suppress viral replication and reduce the risks of cirrhosis, HCC, and liver-related mortality, but they rarely achieve functional cure characterized by hepatitis B surface antigen loss. The persistence of covalently closed circular DNA (cccDNA) remains a major hindrance in HBV eradication. Therefore, novel therapeutic strategies targeting different stages of the viral life cycle, including capsid assembly modulators, small interfering RNAs, nucleic acid polymers, and cccDNA-directed approaches, are under active investigation. This review summarizes the epidemiology, prevention, current therapies, clinical outcomes, and emerging therapeutic advances in HBV infection, highlighting ongoing efforts toward achieving a functional cure and global HBV elimination.

## 1. Introduction

Hepatitis B virus (HBV) infection is one of the most significant global public health challenges of the 21st century. According to the World Health Organization, approximately 254 million people worldwide were living with chronic HBV infection in 2022, corresponding to a global prevalence of roughly 3.2%, with an estimated 1.1 million HBV-related deaths annually [1,2]. The burden of disease is disproportionately concentrated in sub-Saharan Africa and parts of East and Southeast Asia, where HBsAg prevalence exceeds 8%, while immigration from endemic regions continues to sustain prevalence in otherwise low-endemic countries such as the United States and those in Western Europe [3].

Chronic hepatitis B virus infection is the leading cause of hepatocellular carcinoma worldwide, accounting for approximately 33% of liver cancer deaths. Up to 40% of untreated patients progress to hepatic cirrhosis, underscoring the urgency of effective prevention and treatment strategies [4,5]. Universal infant HBV vaccination has been the principle of prophylaxis, which was first adopted in the United States in 1991 and now implemented in 98% of countries globally, reducing HBsAg prevalence among children under 5 years from 4.7% to less than 1% [1,6]. However, gaps in birth-dose coverage, particularly in resource-limited settings and suboptimal adult vaccination rates, remain critical barriers to elimination [1,7].

Current antiviral therapies, i.e., pegylated interferon alfa and nucleoside analogs (entecavir, tenofovir disoproxil fumarate, and tenofovir alafenamide), effectively suppress HBV replication and reduce progression to cirrhosis and hepatocellular carcinoma, with increased survival [8]. Although combination regimens hold the greatest promise for achieving functional cure, most remain in early-phase clinical trials and questions regarding optimal regimens, durability of response, and safety persistance [9].

Even so, the treatments we have almost never clear the infection, and the reason lies in the biology of the virus rather than in the drugs themselves. Three problems which are linked explain why suppressing the virus does not cure it. The first is covalently closed circular DNA (cccDNA). This form of the viral genome resides in the nucleus of infected liver cells as a stable mini-chromosome. Nucleoside analogs cannot act on it, and it can be topped up from within the cell by recycling relaxed circular DNA [10]. The second is integrated HBV DNA. Pieces of the viral genome become part of the host chromosome and keep producing HBsAg on their own, so antigen can still be made when viral replication has been shut down completely. In hepatitis B e antigen (HBeAg)-negative infection, integrated DNA is in fact the main source of circulating HBsAg [11]. The third is the state of the immune system. Long exposure to a large excess of viral antigen wears out and depletes HBV-specific T cells and weakens B-cell responses, so the patient cannot clear the remaining infected liver cells [12]. These three barriers define the problem that new drugs are trying to solve. They also explain why suppressing HBV in the blood, however completely, is not the same as curing the infection.

This review has three aims. The first is to describe the current global epidemiology of HBV infection using primary surveillance data, modeling studies, and guidelines rather than secondary sources. The second is to review established prevention and treatment strategies together with their outcomes and their weaknesses. The third is to give a critical account of new therapies rather than a simple list of them. For each drug class, we describe the molecular target, the reasoning behind it, the reason it cannot achieve functional cure on its own, and the combination approaches that follow from that limitation. Section 2 and Section 3 cover epidemiology and prevention, Section 4 covers current treatment and clinical outcomes, Section 5 covers new therapeutic directions, and Section 6 sets out the main conclusions and the questions that remain open.

## 2. Epidemiology

Hepatitis B virus (HBV) infection remains one of the most significant global public health challenges. According to a survey conducted in 2022 by World Health Organization (WHO), approximately 254 million people worldwide were living with chronic HBV infection, corresponding to a global prevalence of about 3.2%, with an annual incidence of approximately 1.2 million new infections and 1.1 million HBV-related deaths [1,2]. Earlier estimates from the Global Burden of Disease Study placed the number of chronically infected individuals at 296 million in 2019, with 820,000 deaths attributed to HBV-related complications [4]. Notably, viral hepatitis accounted for 1.34 million deaths in 2015, a toll comparable to tuberculosis and exceeding that of human immunodeficiency virus (HIV), with 66%, or roughly two-thirds, of these deaths caused by HBV [8]. HBV-related cirrhosis resulted in an estimated 331,000 deaths in 2019, while HBV-related liver cancer (hepatocellular carcinoma) caused approximately 192,000 deaths, an increase from 156,000 in 2010 [3].

**Figure 1:**
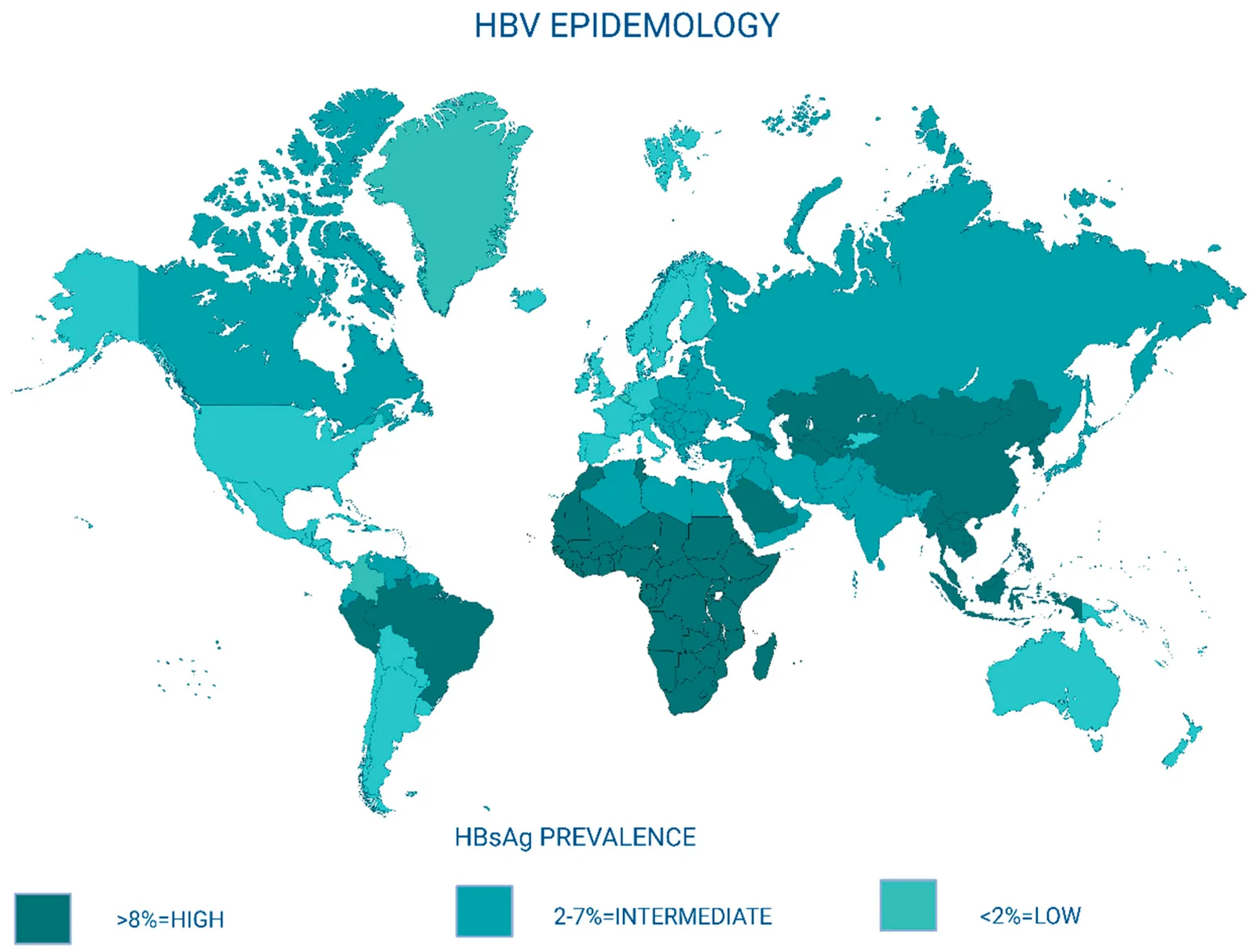
Global epidemiology of HBV infection. World map illustrating the geographic distribution of hepatitis B surface antigen (HBsAg) prevalence. Regions are categorized as high endemicity (>8%), intermediate endemicity (2–7%), and low endemicity (<2%). The highest burden is observed in sub-Saharan Africa and parts of East and Southeast Asia, whereas North America, Western Europe, and Australia are generally low-endemic regions. Prevalence categories are based on WHO regional HBsAg seroprevalence estimates for 2022 [1] and modeled country-level estimates from the Polaris Observatory for the same year [2].

The geographic distribution of HBV infection varies considerably. Endemicity is classified as high (HBsAg prevalence ≥8%), intermediate (2–7%), or low (<2%), as mentioned in Figure 1 [1]. Sub-Saharan Africa bears the highest HBsAg seroprevalence at 8.83%, followed by the Western Pacific region at 5.26% [2]. China, despite not having the highest prevalence (5.49%), accounts for approximately one-third of all chronic HBV infections worldwide due to its large population [2]. Globally, HBV endemicity is geographically distributed in an inverse proportion to income level. All hyperendemic countries (where HBV prevalence is >8%) are low-income or lower middle-income economies as defined by the World Bank, and most of them are “least developed countries” according to the United Nations. The disparity is also evident in Europe, where the prevalence of HBV infection is much lower than 1% in Western and Northern regions but reaches 4–8% in several middle-income Eastern European countries or regions [3]. In contrast, North America, Western Europe, and Australia are classified as low-endemic regions, although prevalence in these areas is influenced by immigration from endemic countries [13,14].

**Figure 2:**
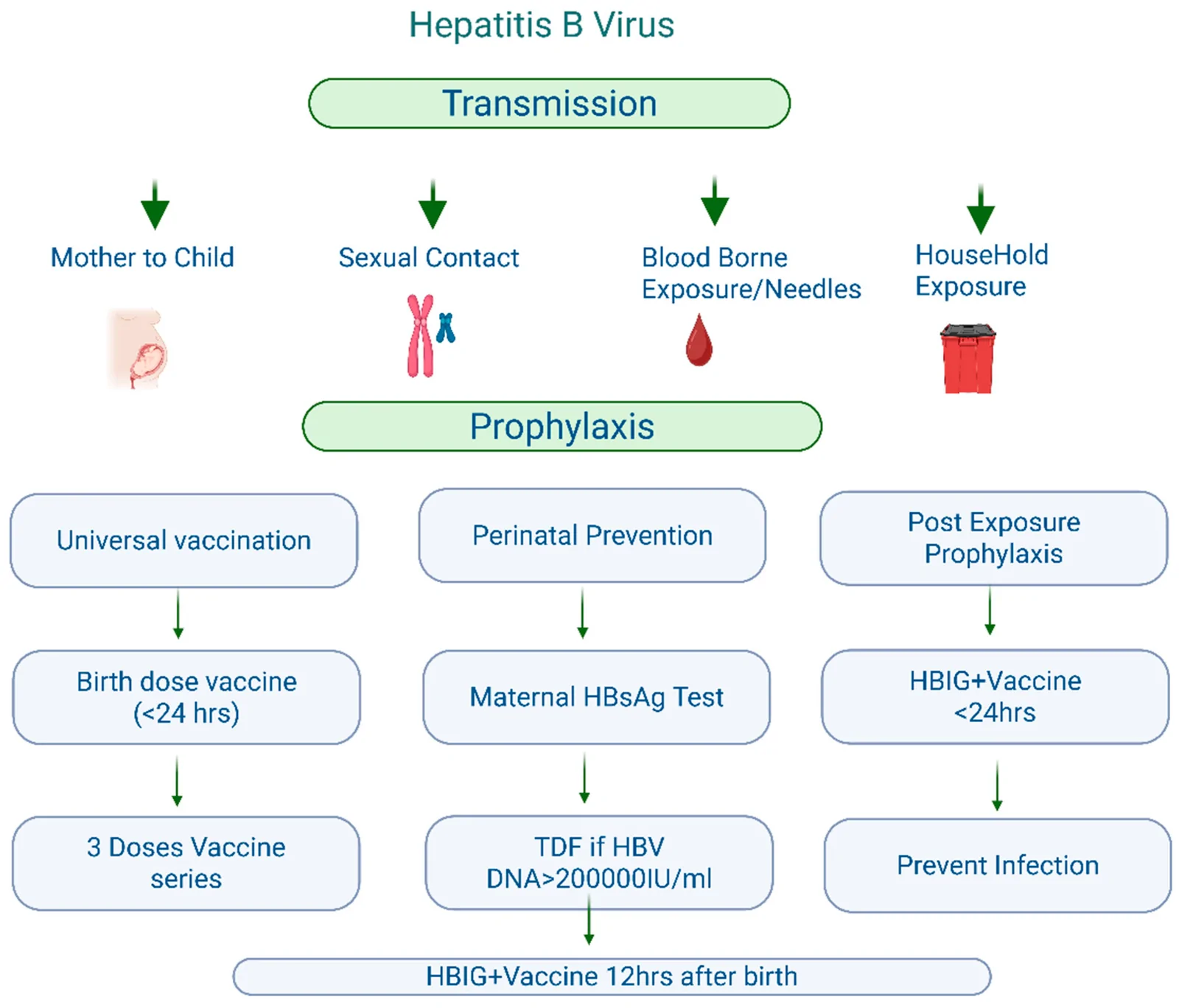
Major routes of HBV transmission and recommended prophylactic strategies. HBV is transmitted through mother-to-child transmission, sexual contact, blood exposure/needle sharing, and household exposure. Prevention includes universal vaccination after birth, completion of the vaccine series, maternal HBsAg screening, tenofovir prophylaxis for mothers with high HBV load, and post-exposure prophylaxis with HBIG and hepatitis B virus vaccination.

HBV is transmitted through percutaneous or mucosal exposure to infected blood and bodily fluids, including semen and vaginal secretions, as mentioned in Figure 2 [13]. The virus is highly stable in the environment, remaining infectious on surfaces for at least 7 days, and it is more infectious than HIV [13]. The predominant modes of transmission vary by region; mother-to-child (vertical) transmission predominates in Asia, sexual exposure and injection drug use predominate in Western countries, and horizontal childhood transmission predominates in Africa [15]. The risk of developing chronic infection is inversely related to age at acquisition. It is approximately 90% when infection occurs in neonates, 20–30% in children under 5 years, and less than 5% in immunocompetent adults [13,16]. In the United States in 2022, the CDC estimated approximately 14,000 new acute HBV infections, with 47% attributed to sexual transmission and 19% to injection drug use [14].

Global coverage of the three-dose infant vaccine series reached 84% in 2024, yet birth-dose coverage remains inadequate at only 45%, with the African region lagging at 17% [1,7]. In countries with early adoption of universal vaccination such as Taiwan, significant reductions in both HBV prevalence and hepatocellular carcinoma incidence have been observed among children and young adults [17]. Despite these advances, the Global Burden of Disease Study 2021 estimated 283.64 million prevalent cases of chronic HBV with CHB-related deaths increasing by 20% from 1990 to 2021, underscoring the urgent need for comprehensive interventions encompassing vaccination, screening, and antiviral therapy to achieve the WHO’s hepatitis B elimination goals by 2030 [18].

## 3. Prophylaxis

Vaccination is the central strategy of HBV prevention. Recombinant HBV vaccines are safe and highly immunogenic, inducing protective levels of antibody to hepatitis B surface antigen (anti-HBs) (≥10 mIU/mL) in over 95% of healthy infants and more than 90% of adults under 40 years of age [13,19]. In 2022, the CDC expanded adult vaccination recommendations to include all persons aged 19–59 years and adults ≥60 years with risk factors or who desire protection [20]. Vaccine-induced immune memory persists for 30 years or more, and booster doses are not routinely required in immunocompetent individuals [16,19].

### 3.1. Global Hepatitis B Vaccination Coverage and Regional Differences

The vaccination policy described above is largely from a United States perspective, so the global picture needs separate attention. Hepatitis B vaccine was added to the WHO Expanded Programme on Immunization in 1992, and by 2024, almost every WHO Member State had put infant hepatitis B vaccination into its national schedule [1]. Global coverage with three doses of hepatitis B-containing vaccine was 84% in 2024, but this single number hides two important differences [7]. The first is the gap between the three-dose series and the timely birth dose. The birth dose is the one step that does most to stop mother-to-child transmission, and global coverage was only about 45% in 2022, roughly half the three-dose figure. The second is the gap between WHO regions. Birth-dose coverage is above 80% in the Western Pacific Region, where universal birth-dose policies have been in place for decades, but only about 17% in the African Region of the same year, the region that also has the highest HBsAg prevalence [1,7]. WHO has since revised these figures downwards, to 43% globally and 11% in the African Region, so the gap described here has widened rather than closed [7]. Because the region with the greatest need has the least coverage, the WHO target of cutting HBsAg prevalence in children under 5 to below 0.1% by 2030 has already been met in some regions while remaining far out of reach in others [1]. Good global averages should therefore not be read as evidence that the program is on track everywhere in the world.

### 3.2. Limitations of Hepatitis B Vaccination

The success rates quoted above apply to healthy people with a normal immune system and do not hold for everyone. About 5% to 10% of healthy adults do not reach an anti-HBs level of 10 mIU/mL after a full three-dose course, and they are described as non-responders [19]. The reasons are well known. They include age over 40 years, male sex, obesity, smoking, diabetes, chronic kidney disease, and alcohol use. Genetic factors, particularly human leukocyte antigen (HLA) class II types, account for much of the remaining variation [21]. Giving a second three-dose course produces protective antibody in about half of those who did not respond the first time. Anyone who is still negative after six documented doses is regarded as a true non-responder and should be treated as susceptible, which means they need HBIG rather than vaccine after an exposure [19,22]. A person who stays negative should also be tested for HBsAg, because undiagnosed chronic infection is an important alternative explanation for what appears to be vaccine failure.

Several other groups respond less well. Patients with end-stage kidney disease on hemodialysis respond poorly to standard schedules and need higher-dose or four-dose regimens, and even then, they reach protective levels less reliably and lose antibody faster than healthy adults. Weaker responses are also seen in people living with HIV, in recipients of solid organ or stem cell transplants, in patients on immunosuppressive or biologic drugs, in people with obesity, and in older adults in whom ageing of the immune system plays a part of its own [19,21]. Newer adjuvants help with this problem. A two-dose vaccine adjuvanted with a Toll-like receptor 9 agonist (HepB-CpG) produced significantly higher protection rates than a standard aluminum-adjuvanted three-dose vaccine in adults, including in people with diabetes and in older recipients [23]. The choice of vaccine and schedule should therefore be tailored to the patient rather than applied uniformly, and post-vaccination antibody testing is recommended in groups at high risk of not responding [19].

A separate concern is the emergence of vaccine escape variants or mutants. Changes in the main antibody target of the S gene, known as the “a” determinant, of which sG145R is the best known, can allow infection even when anti-HBs are present at levels normally regarded as protective. Some of these variants are also missed by certain HBsAg tests. Escape variants have been found in vaccinated infants born to HBsAg-positive mothers and in liver transplant recipients given HBIG. However, 40 years of universal vaccination have not shown these variants replacing wild-type virus or reducing how well vaccination programs work, and their frequency has stayed low and stable in most surveillance studies [24]. The sensible conclusion is that molecular surveillance should continue, not that current vaccines are failing.

### 3.3. Adult Vaccination Outside the United States

The 2022 recommendation of the Advisory Committee on Immunization Practices (ACIP) to vaccinate all adults aged 19 to 59 years was a deliberate move away from risk-based vaccination. Risk-based programs had consistently underperformed because they depend on doctors asking about, and patients admitting to, behaviors that carry stigma, and adult coverage in the United States had stayed at around 30% [20]. Applying this policy worldwide is less simple than it looks. Universal adult vaccination is most cost-effective where a large number of adults are still susceptible, where adult infections are not rare, and where vaccine and delivery costs are low relative to the health budget. These conditions do not apply everywhere. In high-burden, low-resource countries, the extra benefit of adult vaccination is generally smaller than the benefit of achieving universal timely birth-dose coverage and better antenatal screening because most chronic infections in those settings are acquired at birth or in early childhood rather than during adult life [1]. The practical barriers are also different in kind. Adult immunization services often do not exist, there is no routine adult health contact equivalent to antenatal or child health visits, funding mechanisms such as Gavi are built around childhood schedules, and adults frequently fail to complete multi-dose courses without reminder systems. For these reasons, WHO continues to give priority to universal infant vaccination with a timely birth dose, together with targeted vaccination of high-risk adults, rather than to universal adult vaccination [1]. Arguments for wider adult vaccination should therefore be based on local epidemiology and health system capacity rather than on direct comparison with United States policy.

### 3.4. Recent Changes in United States Vaccination Policy

The United States is used here as a case study rather than as a special case because it is the setting in which the effect of withdrawing a universal birth-dose recommendation has actually been measured, and the lesson may apply to any country considering a similar change.

The possibility of making the HBV vaccination optional and reducing vaccine uptake is documented and is a major clinical concern. In 1999, the American Academy of Pediatrics and the US Public Health Service recommended delaying birth-dose vaccination for infants of HBsAg-negative mothers due to thimerosal concerns. Coverage among infants of unscreened mothers fell rapidly from 53% to 7%, returning only after universal birth-dose vaccination was suspended. Given that 12% to 16% of pregnant women remain unscreened despite CDC recommendations, even modest declines in birth-dose coverage among this group may meaningfully increase neonatal infections [25].

Even before the December 2025 ACIP decision, US newborn HBV vaccination rates had already declined by more than 10% over the preceding 2 years, coinciding with increased public discourse regarding childhood vaccination following the COVID-19 pandemic [26].

Multiple modeling studies published in 2026 have estimated the expected consequences of the ACIP policy change. Lind et al. developed a compartmental model assessing the impact of replacing universal birth-dose vaccination with the targeted shared clinical decision-making (SCDM) approach [25]. Under the universal recommendation, the model estimated a median of 1292 neonatal HBV infections per birth cohort. Under the targeted recommendation, if birth-dose coverage among infants of unscreened mothers declined to 10%, much like the decline seen when universal vaccination was temporarily removed in 1999, the model projected approximately 628 additional neonatal infections and 565 additional chronic infections per birth cohort [25].

An economic evaluation demonstrated that delaying the first HBV vaccine dose to 2 months for infants of parents with unknown HBsAg status would result in approximately 1400 excess new HBV infections and over USD 222 million in additional healthcare costs [27].

### 3.5. Perinatal Prophylaxis

Prevention of mother-to-child transmission is a critical component of HBV elimination strategies. All pregnant women should be screened for HBsAg, and infants born to HBsAg-positive mothers should receive both HBV vaccine and hepatitis B immune globulin (HBIG) within 12 h of birth [19,28]. For mothers with high viral load (HBV DNA > 200,000 IU/mL), maternal antiviral prophylaxis with tenofovir disoproxil fumarate (TDF) during the third trimester significantly reduces the risk of vaccine failure and perinatal transmission [8]. A meta-analysis confirmed that maternal antiviral therapy approximately halves the rate of HBsAg seropositivity in newborns compared with immunoprophylaxis alone [29].

The maternal HBV DNA threshold of 200,000 IU/mL, which is about 5.3 log10 IU/mL, is often quoted without explanation, so it is worth describing where it comes from. The risk that vaccine and HBIG will fail rises with maternal viral load in a continuous but uneven way. Transmission despite immunoprophylaxis is very rare, below about 200,000 IU/mL, and then it climbs steeply, reaching 8% to 15% when maternal HBV DNA is above 8 log10 IU/mL. The threshold is therefore not a biological cut-off but a practical operating point, chosen at the level where the absolute risk of failure becomes high enough to justify giving antiviral drugs in the third trimester. The American Association for the Study of Liver Diseases (AASLD), the European Association for the Study of the Liver (EASL), and WHO have all adopted it on that basis [16,30,31]. Where HBV DNA testing is not available, maternal HBeAg status is a workable substitute, and WHO specifically recommends an HBeAg-based approach for this reason [32].

Tenofovir disoproxil fumarate (TDF) is the preferred drug and is started at 28 to 32 weeks of pregnancy. This timing gives the largest reduction in viral load before delivery while keeping the length of fetal drug exposure short [30]. The evidence requires careful analysis. In a randomized trial in China of mothers with HBV DNA above 200,000 IU/mL, TDF started at weeks 30–32 reduced transmission from 7% to 0% in the per-protocol analysis [33]. By contrast, the placebo-controlled iTAP trial in Thailand found no significant benefit from TDF because transmission with vaccine and HBIG alone was already only 2% [34]. These results do not contradict each other. They show that the benefit of maternal antiviral treatment depends on how good neonatal immunoprophylaxis is. Where HBIG is given promptly and the vaccine course is completed, the extra gain from TDF is small. Where HBIG is unavailable, unaffordable, or delayed, which describes much of sub-Saharan Africa, maternal antiviral treatment becomes correspondingly more important. Treatment is usually continued until delivery or for 4 to 12 weeks afterwards, and alanine aminotransferase (ALT) should be monitored for several months after stopping because a minority of women develop a hepatitis flare after giving birth [30]. Newer data support the safety and effectiveness of tenofovir alafenamide in pregnancy, although experience with it is still more limited than with TDF [35].

Mode of delivery and infant feeding often cause uncertainty in practice. Elective caesarean section is not recommended simply to prevent HBV transmission. The observational evidence is inconsistent and is confounded by the reason for the caesarean and by maternal viral load, and no randomized study has shown a benefit once effective immunoprophylaxis and, where needed, maternal antiviral treatment are in place [30]. Invasive procedures such as amniocentesis are not absolutely contraindicated but are best avoided when the maternal viral load is high. Breastfeeding is safe in HBsAg-positive mothers whose infants have received timely immunoprophylaxis. A meta-analysis found no increase in transmission in breastfed compared with formula-fed infants [36], and both AASLD and WHO support breastfeeding in this situation [30,32]. Mothers with cracked or bleeding nipples should stop feeding from the affected breast until it has healed. Breastfeeding is also compatible with maternal TDF treatment.

Post-vaccination serologic testing (PVST) completes the care of these infants and is often omitted. Infants born to HBsAg-positive mothers should be tested for HBsAg and anti-HBs at 9 to 12 months of age, or 1 to 2 months after the last vaccine dose if the course has been delayed. Anti-HBc should not be used because antibody passed from the mother can persist for up to 24 months [19]. An infant who is HBsAg-negative with anti-HBs titers of 10 mIU/mL or more is protected and needs nothing further. An infant who is HBsAg-negative with anti-HBs titers below 10 mIU/mL should be given a second three-dose course and retested 1 to 2 months after the last dose. An infant who is HBsAg-positive has been infected despite prophylaxis and should be referred to a pediatric liver service for long-term follow-up [19,28].

### 3.6. Post-Exposure Prophylaxis

For unvaccinated individuals with percutaneous, mucosal, or sexual exposure to an HBsAg-positive source, combined administration of HBIG and initiation of the HBV vaccine series is recommended ideally within 24 h of exposure [16]. Previously vaccinated individuals with documented seroprotection generally require no additional intervention [28]. Universal screening with HBsAg, anti-HBs, and anti-HBc is recommended before initiating immunosuppressive or anticancer therapy [37].

Post-exposure prophylaxis is easiest to organize and understand around three questions. What kind of exposure occurred, is the source HBsAg-positive, and what is the vaccination and antibody status of the exposed person? Together, these determine what should be administered. In every case, prophylaxis should be started as early as possible. HBIG works best within 24 h and is of doubtful value beyond 7 days after an exposure through the skin, or beyond 14 days after sexual exposure [19,22].

For occupational exposure through the skin or a mucous membrane, such as a needlestick injury or a splash onto mucous membranes or broken skin, the source should be tested for HBsAg, and the exposed worker should be assessed for immunity. Staff who have completed a vaccine course and have a documented anti-HBs titers of 10 mIU/mL or more at any point in the past need nothing further, no matter the source status. Staff who have completed a vaccine course but have no documented antibody level should have anti-HBs titers measured. If the titer is 10 mIU/mL or more, no action is needed. If it is below 10 mIU/mL and the source is HBsAg-positive or of unknown status, the person should be given HBIG at 0.06 mL/kg together with a booster dose of vaccine, and anti-HBs should be rechecked 1 to 2 months later. Individuals who are unvaccinated or partly vaccinated should be given HBIG and should start or complete the vaccine course. A person who is a documented non-responder after two full courses should be given two doses of HBIG 1 month apart after exposure to an HBsAg-positive or unknown source because further vaccination is unlikely to help [19,22].

For sexual or household exposure to someone with acute or chronic HBV infection, unvaccinated contacts should be given a single dose of HBIG together with the first dose of vaccine, ideally within 14 days of the last sexual contact, and they should then complete the course. Contacts who have been vaccinated and have documented protective antibody need nothing further. Contacts whose response is unknown should be given a booster dose of vaccine, and HBIG should be added if the source is HBsAg-positive and the contact is known or thought not to have responded. The same approach applies after sharing injecting equipment. If the source cannot be tested, the exposure should be managed as though the source were HBsAg-positive whenever there is any epidemiological risk [16,19]. People who have been sexually assaulted should be vaccinated, with HBIG added if the assailant is known to be HBsAg-positive.

Separately from managing acute exposures, everyone should be screened with HBsAg, anti-HBs, and anti-HBc before starting immunosuppressive, anticancer, or anti-CD20 treatment. The risk of reactivation depends on the pattern of these markers and on the drug regimen rather than on any history of exposure [37].

### 3.7. Adapting Prevention to High-Burden, Low-Resource Regions

The differences described in Section 3.1 will not be closed by applying strategies designed for wealthy countries because the barriers in high-burden regions are structural rather than a matter of attitude. In much of sub-Saharan Africa, the main obstacle to birth-dose coverage is that many births take place outside health facilities or are attended by traditional birth attendants, so the newborn never reaches the health system within the 24 h window. Other constraints include limited cold-chain capacity in rural districts, vial wastage rules that discourage opening a multi-dose vial for one newborn, incomplete antenatal HBsAg screening, and the cost and near-absence of HBIG [1,32]. Several practical adaptations follow from these constraints. Monovalent hepatitis B birth dose can be delivered in a controlled temperature chain, which allows limited storage outside the cold chain and lets trained community health workers give it, including for at home births. Because HBIG is often simply unobtainable, WHO recommends a strategy built on universal timely birth-dose vaccination plus maternal tenofovir based on HBeAg status, or on HBV DNA where testing exists, rather than on HBIG [32]. Adding HBsAg screening to antenatal services that already exist for HIV and syphilis, using point-of-care rapid tests, avoids the need for laboratory infrastructure and allows same-day results. Simplified treatment criteria that do not require HBV DNA testing or elastography, together with shifting tasks to non-specialist clinicians and moving care into primary care, address the parallel bottleneck in access to treatment [32]. In much of Asia, the binding constraint is different. Birth-dose coverage is already high, and the problem is finding and treating the large number of adults who were infected before the vaccine was introduced. There, the priority is population screening, liver cancer surveillance, and scaling-up treatment rather than further work on infant vaccination [1,2]. Elimination strategy should therefore differ by region according to which step in the pathway from prevention to treatment is the rate-limiting one.

## 4. Therapy

The primary objectives of antiviral therapy for chronic hepatitis B (CHB) are prevention of progression to cirrhosis, hepatic decompensation, hepatocellular carcinoma (HCC), and liver-related death [13,38]. Because these clinical endpoints take years or decades to develop, surrogate measures are used to assess treatment response: virological (undetectable HBV DNA by polymerase chain reaction (PCR)-based assay), serological (HBeAg loss and seroconversion, HBsAg loss and seroconversion), biochemical (ALT normalization), and histological (histologic improvement in necroinflammation by ≥2 points without worsening fibrosis) [38]. HBsAg loss, termed “functional cure,” is the optimal endpoint and is associated with improved long-term prognosis, although it occurs at very low rates with current therapies [39].

### 4.1. What Cure Means and Why HBsAg Loss Is an Imperfect Endpoint

The word cure is used loosely in the hepatitis B literature. The available endpoints differ a great deal in what they mean about the virus remaining in the liver, so clear terms are required before clinical trial results can be interpreted. Guidance from the joint EASL-AASLD treatment endpoints conference distinguishes three [40]. Complete cure, also called sterilizing cure, means eliminating all forms of the virus, including cccDNA in the nucleus and HBV DNA integrated into the host genome, with loss of HBsAg and development of high titer anti-HBs. No current or experimental treatment achieves this, and no ongoing phase 3 program uses it as an endpoint. Functional cure, the endpoint used in current trials, means lasting loss of HBsAg, with or without anti-HBs, together with undetectable HBV DNA, measured at least 24 weeks after a fixed course of treatment has ended. In this state, cccDNA is still present but is transcriptionally silent. This is the same state reached by healthy adults who clear acute hepatitis B on their own, so it is known to be biologically possible. Partial cure means HBV DNA stays undetectable off treatment while HBsAg remains positive [39].

The limitations of HBsAg loss as an endpoint follow from this. Because cccDNA and integrated HBV DNA remain after HBsAg has cleared, functional cure is a state of immune control rather than eradication, and this has three practical consequences. First, the virus can reactivate when immune control is removed, most obviously during B-cell-depleting or intensive immunosuppressive treatment, so patients who have cleared HBsAg still need risk assessment and sometimes prophylaxis [37]. Second, the risk of liver cancer falls a great deal after HBsAg loss but does not disappear, particularly in patients who already have cirrhosis or who clear HBsAg at an older age, so surveillance should not be stopped on the basis of HBsAg loss alone [38,41]. Third, quantitative HBsAg is an imperfect measure of how much virus remains in the liver. In the HBeAg-negative disease, much of the circulating antigen comes from integrated HBV DNA and therefore does not track cccDNA activity, which means a drug can lower HBsAg substantially without changing the cccDNA pool at all, and the reverse is also possible [11]. Trials that use HBsAg loss as their primary endpoint should be interpreted with these points in mind.

Other markers are being studied to get around these limitations. Hepatitis B core-related antigen (HBcrAg) combines HBeAg, HBcAg, and the p22cr protein in a single measurement. It tracks cccDNA and intrahepatic HBV DNA more closely than HBsAg does, and it falls more steeply in patients who go on to achieve functional cure or lose HBeAg. Serum HBV pregenomic RNA reflects how active cccDNA transcription is and stays detectable during nucleoside analog treatment even when HBV DNA is fully suppressed, which makes it a candidate for predicting relapse after treatment stops. A low baseline quantitative HBsAg, usually below 100 to 1000 IU/mL depending on the regimen, is the most consistently reproduced predictor of later HBsAg loss across interferon-based and RNA-based trials. None of these markers is yet validated as a replacement endpoint. Their main value at present is in choosing patients and in judging how durable an off-treatment response will likely be.

### 4.2. Nucleoside Analog Therapy

Nucleoside analogs (NAs) represent the mainstay of CHB treatment worldwide. Three preferred first-line agents, entecavir (ETV), tenofovir disoproxil fumarate (TDF), and tenofovir alafenamide (TAF), provide potent antiviral activity, high barriers to resistance, and favorable tolerability profiles [8]. These agents act as chain terminators that inhibit the enzymatic activity of HBV polymerase, blocking negative-strand DNA synthesis [42]. Older Nas, including lamivudine, adefovir, and telbivudine have been rendered obsolete due to inferior resistance profiles [8].

**Figure 3:**
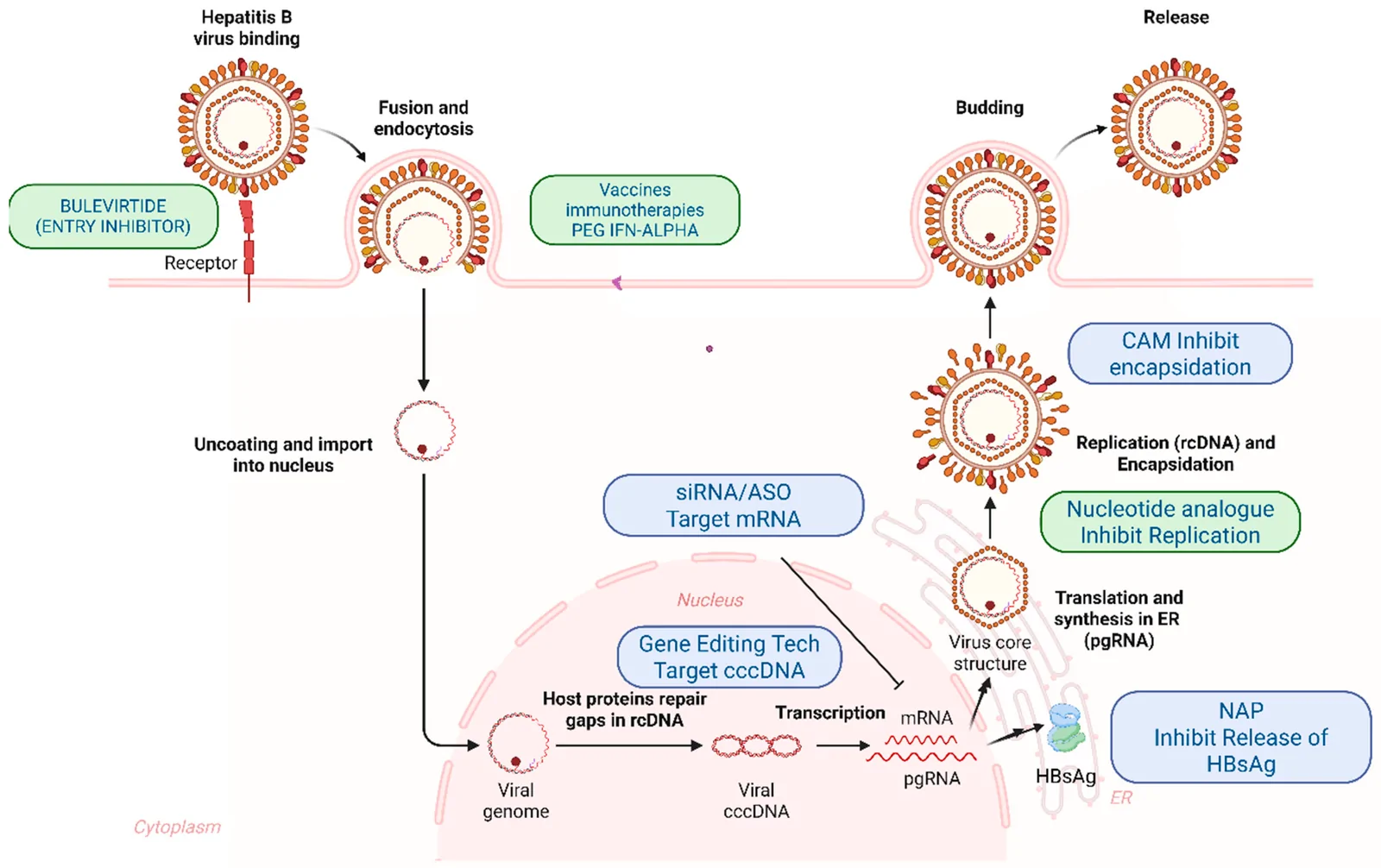
HBV life cycle and therapeutic targets. This illustrates HBV entry, uncoating, cccDNA formation, transcription, translation, replication, encapsidation, budding, and viral release. Current (green boxes) and emerging therapies (blue boxes) target different stages of the viral life cycle, including entry inhibitors (bulevirtide), nucleoside analogs, capsid assembly modulators (CAMs), siRNA/antisense oligonucleotides, nucleic acid polymers (NAPs), immunotherapies, and gene-editing approaches directed against cccDNA.

In HBeAg-positive patients, 1 year of NA therapy achieves HBV DNA suppression in 64–76%, ALT normalization in 68–72%, histological improvement in 49–74%, HBeAg seroconversion in 10–21%, and HBsAg seroclearance in only 1–3% [14]. In HBeAg-negative patients, 1-year HBV DNA suppression rates are higher at 90–94%, with ALT normalization in 76–83% and histological improvement in 60–72%, but HBsAg seroclearance remains below 1% [14]. Long-term treatment extending to 7–10 years maintains HBV DNA suppression in 95–98% of patients, with cumulative HBeAg seroconversion increasing to 27–38%, though HBsAg loss remains at only 3–5% (Figure 3 [13,14]).

### 4.3. Comparative Efficacy

Meta-analyses comparing TDF and ETV in treatment-naïve CHB patients have demonstrated comparable efficacy in HBV DNA suppression, ALT normalization, and HBeAg seroconversion at 48 weeks [43,44]. A large multicenter REAL-B study, including 1605 patients across 22 international centers, found that 5-year cumulative virological response rates were high in both groups, with a modestly higher rate in TAF patients vs. ETV patients (98.0% vs. 93.9%), while biochemical response rates were similar (93.7% vs. 92.8%) [45]. A Korean multicenter study of 4210 patients demonstrated comparable outcomes between ETV and TDF regarding HCC incidence, death or liver transplantation, decompensation events, and complete virological response rates (95.1% vs. 95.8%) [46]. Selection among these agents should be guided by cost, side-effect profile, and patient-specific factors, including renal function, bone health, pregnancy status, and HIV coinfection [45].

### 4.4. Pegylated Interferon Alpha

Pegylated interferon alfa (PEG-IFN) is given as a fixed 48-week course by subcutaneous injection and works in two ways: directly against the virus and by modifying the immune response. Its effect on cccDNA is indirect, and describing PEG-IFN as causing cccDNA degradation is too simple, because at least three separate mechanisms have been described. First, interferon alfa switches on interferon-stimulated genes whose products block several steps of the viral life cycle, including encapsidation and the stability of viral RNA. Second, interferon alfa reduces transcription from the cccDNA minichromosome. It lowers the binding of transcription factors to cccDNA-bound histones and causes hypoacetylation of histones H3 and H4 along with recruitment of transcriptional repressors. This epigenetic silencing has been shown in cell culture and in HBV-infected humanized mice and does not involve loss of the cccDNA template itself [47]. Third, there is a degradative pathway that does not kill the cell. Interferon alfa increases the cytidine deaminases APOBEC3A and APOBEC3B, which are brought to cccDNA through the viral core protein and cause cytidine deamination, followed by breakdown of the cccDNA without the hepatocyte dying [48]. Interferon also strengthens innate and adaptive immune responses, which helps clear infected liver cells [49]. How much each of these mechanisms contributes in treated patients is not known, and the fall in cccDNA seen in practice is modest. Writing that PEG-IFN eliminates cccDNA is therefore not supported by the clinical evidence.

A 48-week course achieves HBeAg seroconversion in approximately 30% and HBsAg seroclearance in 3–4% of HBeAg-positive patients [13]. In HBeAg-negative patients, sustained virological response (HBV DNA < 2000 IU/mL) is observed in up to 40%, with HBsAg loss reported in approximately 12% at 5 years post-treatment [49]. Real-world data from the S-Collate study confirmed that on-treatment HBsAg kinetics predict 3-year HBsAg clearance, with HBsAg levels < 1500 IU/mL at week 12 associated with an 11% clearance rate in HBeAg-positive patients [50]. PEG-IFN use is limited by significant adverse effects, including myelosuppression and thyroid dysfunction; it is contraindicated in decompensated cirrhosis, pregnancy, and autoimmune diseases [13].

### 4.5. Impact on HCC Risk

**Figure 4:**
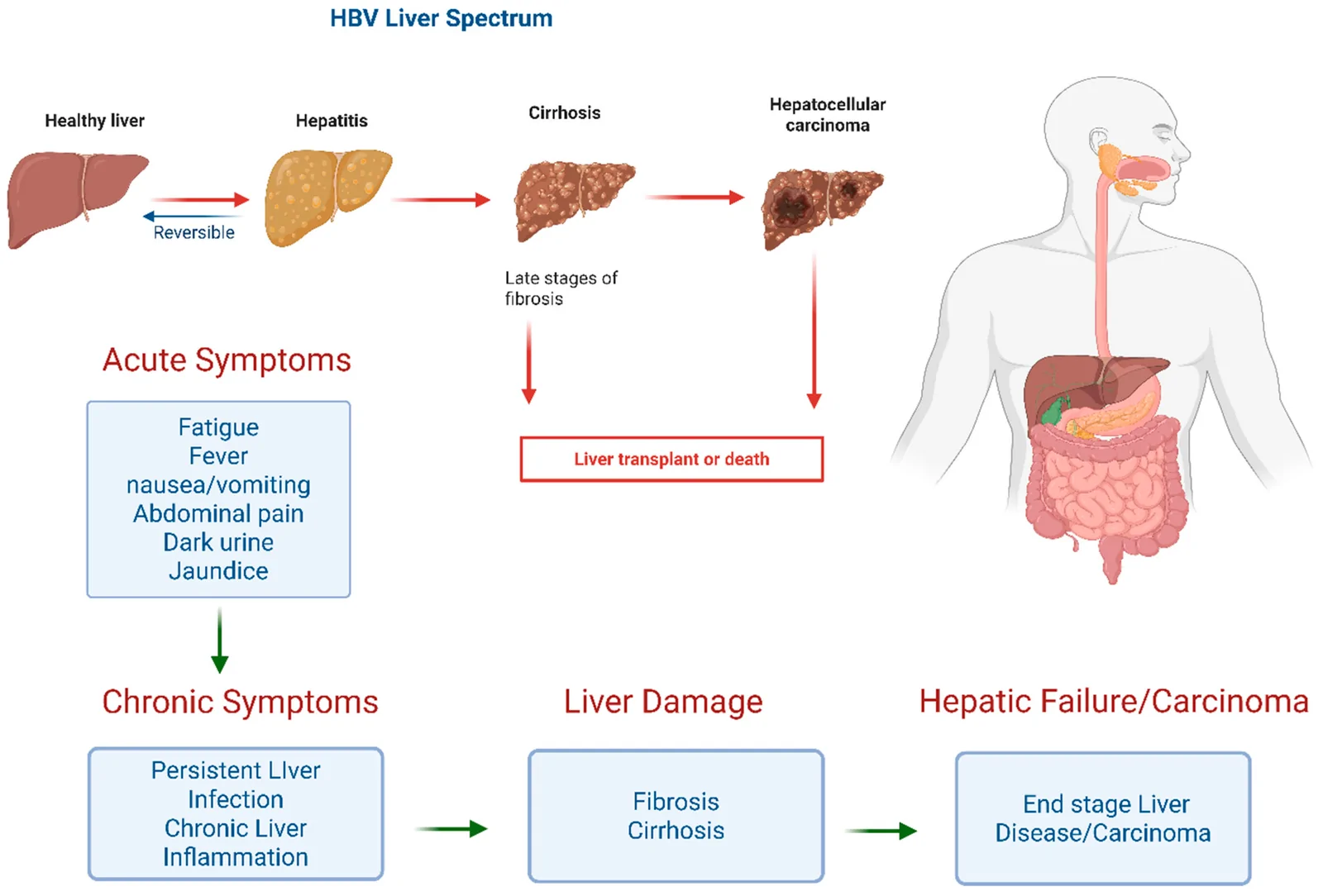
Clinical spectrum and progression of disease caused by HBV. HBV infection may progress from acute hepatitis to chronic infection, leading to persistent liver inflammation, fibrosis, cirrhosis, hepatocellular carcinoma, liver failure, and death. Early stages may be reversible, while advanced liver disease often requires liver transplantation. Common acute manifestations include fatigue, fever, nausea, vomiting, abdominal pain, dark urine, and jaundice. And different Colour arrows Function same in figure.

Antiviral therapy substantially reduces HCC risk. A systematic review and meta-analysis demonstrated that long-term NA therapy was associated with pooled relative risks of 0.57 in patients with cirrhosis and 0.50 in those without cirrhosis, compared with untreated individuals [14]. Annual HCC incidence rates in NA-treated patients range from 0.01–1.4% in non-cirrhotic patients and 0.9–5.4% in those with cirrhosis [41]. A network meta-analysis found that both ETV and TDF significantly lowered HCC risk compared with older NAs (HR 0.60 and 0.56, respectively), with no significant difference between the two agents [51]. Importantly, antiviral therapy also reduces HCC risk in patients in the indeterminate phase, with a 70% reduction demonstrated in a multicenter study (adjusted HR 0.3, 95% CI 0.1–0.6) [52]. The ATTENTION trial provided randomized evidence supporting early antiviral treatment with TAF in non-cirrhotic CHB patients with moderate or high viral-load, irrespective of ALT concentrations, to prevent HCC and other serious clinical events [53]. Despite these benefits, the risk of HCC persists, demanding continued surveillance particularly in patients with cirrhosis (Figure 4) [14].

### 4.6. Widening the Indications for Antiviral Treatment

The most important recent change in chronic hepatitis B is not a new drug but a change in who should be treated, and it deserves discussion in its own right. Conventional criteria combine ALT level, HBV DNA level, and evidence of fibrosis, and that leaves a large group of patients ineligible for therapy. Between a quarter and a third of patients in long-term cohorts do not fit neatly into any classical phase and are labeled indeterminate or grey zone. These patients have usually been watched rather than treated on the assumption that their risk is similar to that of inactive carriers [38].

The ATTENTION trial then provided randomized evidence, showing that early treatment with tenofovir alafenamide in non-cirrhotic patients with moderate or high viral load reduced serious clinical events, regardless of the ALT level [51]. At the same time, the 2024 WHO guidelines widened eligibility considerably. They recommend treatment for adults with significant fibrosis on non-invasive testing no matter the ALT or HBV DNA level, for those with persistently abnormal ALT and HBV DNA above 2000 IU/mL, whatever the fibrosis stage, and for defined groups, including people with HIV or hepatitis D coinfection, a family history of liver cancer, or relevant comorbidities. They also allow simplified criteria that do not need HBV DNA testing, specifically so that treatment can be scaled up where laboratory capacity is limited [32]. The AASLD and EASL documents have moved in the same direction [31,37].

This agreement between guidelines should still be examined critically rather than simply accepted. The evidence rests on interim results from a single trial, and the cohort data-supporting treatment of indeterminate-phase patients are observational and open to confounding by indication despite propensity adjustment. Wider eligibility also commits many more people to lifelong treatment, with higher associated costs, adherence demands, and monitoring burden, and with the long-term kidney and bone considerations that apply to tenofovir disoproxil fumarate. The alternative is not free of cost either, since it means lifelong surveillance of untreated patients, and the balance will differ between health systems. What can be said with confidence is that the old threshold for treatment was set too high for a meaningful group of patients, and that every major guideline is now moving towards earlier treatment [32,38,53].

### 4.7. Prevention of Mother-to-Child Transmission (MTCT)

Mother-to-child transmission (MTCT) remains the principal cause of chronic HBV infection globally, occurring in 70–90% of infants born to HBeAg-positive mothers without intervention [14]. Antiviral prophylaxis with TDF initiated at 28–32 weeks of gestation in women with HBV DNA > 200,000 IU/mL reduces MTCT from approximately 18% to 5% when combined with neonatal immunoprophylaxis [30,35]. When maternal antiviral therapy is combined with passive–active immunoprophylaxis, the risk of MTCT may be reduced to less than 1% [14]. TDF remains the preferred antiviral agent in pregnancy, though emerging data support the safety and efficacy of TAF during pregnancy [35].

## 5. Future Directions and Novel Therapeutic Strategies for HBV

### 5.1. Pursuit of Functional Cure

Current approved therapies for CHB are pegylated interferon alfa and nucleoside analogs (NAs). These effectively suppress HBV replication but rarely achieve functional cure, defined as sustained undetectable HBsAg and HBV DNA at least 24 weeks after cessation of finite-duration treatment [13]. Functional cure occurs in only up to 7% of patients after 12 months of standard treatment [54]. The persistence of covalently closed circular DNA (cccDNA) and integrated HBV DNA, coupled with impaired host immune responses, remains the principal barriers to viral eradication [12].

### 5.2. Directly Acting Antivirals Inhibiting Viral Replication

Each class of direct-acting antiviral (DAA) in development blocks a particular step of the HBV life cycle (Figure 3). These steps stand in different relationships to the cccDNA reservoir and to HBsAg production, so the classes differ in what they can and cannot achieve. Each class is therefore discussed below in terms of its molecular target, the reasoning behind it, the clinical evidence, and the specific reason it cannot produce functional cure on its own.

#### 5.2.1. Entry Inhibitors

Bulevirtide is a myristoylated 47-amino-acid lipopeptide copied from the preS1 region. It binds NTCP and competitively blocks the virus from attaching and entering. The strongest clinical evidence is in chronic hepatitis D. In a phase 3 randomized trial, 48 weeks of bulevirtide at 2 mg or 10 mg daily produced a combined virological and biochemical response in about 45% and 50% of patients, compared with 2% in those given no antiviral treatment [55]. Responses were maintained or improved over 144 weeks of treatment but fell substantially after the medication was stopped, with only about a third of patients still having a virological response after 96 weeks of follow-up [56]. Data in HBV infection without HDV are still limited [57]. The limitation of this class is decisive and follows directly from the target. Blocking NTCP stops the virus infecting new liver cells and stops it spreading from cell to cell, but it does nothing to cccDNA that is already inside infected cells, nothing to integrated HBV DNA, and nothing to the making and release of HBsAg from either of them. In established chronic infection, cccDNA is already present in a large proportion of hepatocytes and those cells turn over slowly, so blocking entry produces little or no decrement in HBsAg and cannot, even in principle, reduce the reservoir. Its most sensible role is as part of a combination, as a way of stopping the liver being reseeded during or after treatment aimed at the reservoir, and in HDV coinfection, where suppressing HDV is the goal in itself.

#### 5.2.2. Capsid Assembly Modulators

Capsid assembly modulators (CAMs), also called core protein allosteric modulators, bind to a hydrophobic pocket where two core protein molecules meet and change how the nucleocapsid assembles [58]. Class I compounds, of which the heteroaryldihydropyrimidines are the best known, push assembly towards abnormal non-capsid polymers. Class II compounds, including the sulfamoylbenzamides and phenylpropenamides, speed up assembly into capsids that look normal but are empty because they have failed to package pregenomic RNA (pgRNA). Both routes stop pgRNA being packaged and so block reverse transcription, which produces a rapid and very large fall in HBV DNA and HBV RNA in the blood. CAMs also interfere with the recycling pathway by which relaxed circular DNA returns to the nucleus and tops up the cccDNA pool, and this dual action has been shown in primary human hepatocytes [58]. In the phase 2b REEF-1 trial, the CAM bersacapavir combined with an siRNA and a nucleoside analog produced large decreases in HBV DNA and HBV RNA, yet loss of HBsAg remained rare, and no regimen produced functional cure in more than a small minority of patients [59].

The gap between the very large reduction in HBV DNA and RNA and the negligible decrease in HBsAg is the key observation about this class. Capsid modulation acts after transcription has already occurred. It stops new infectious nucleocapsids being produced, but it does not remove the cccDNA template, silence it, or stop it from producing the subgenomic transcripts that encode the surface proteins. More importantly, it has no effect at all on HBsAg made from integrated HBV DNA, which is the main source of circulating surface antigen in HBeAg-negative patients [11]. Blocking the replenishment of cccDNA is a real effect but matters less than it might seem in established infection because in a chronically infected liver, the cccDNA pool is maintained chiefly by the long life of the minichromosome and of the infected cell itself rather than by replenishing from within the cell. Blocking replenishment therefore stops the reservoir growth but does not make it smaller. CAMs reduce how much virus the infection produces without reducing the reservoir that sustains it, so they cannot achieve functional cure on their own.

#### 5.2.3. Small Interfering RNAs and Antisense Oligonucleotides

RNA-based medications act after transcription and are the only current class that lowers circulating HBsAg substantially. Small interfering RNAs (siRNAs), including xalnesiran, JNJ-73763989, and VIR-2218, are attached to N-acetylgalactosamine so that liver cells take them up through the asialoglycoprotein receptor, and they then direct RISC-mediated cleavage of HBV transcripts. The antisense oligonucleotide (ASO) bepirovirsen works differently, triggering RNase H-dependent breakdown of HBV RNA, and it also has immune-stimulating activity through TLR8. The clinical activity is substantial by the standards of this field. Xalnesiran, with or without an immunomodulator, produced durable falls in HBsAg in the phase 2 Piranga trial, with HBsAg loss maintained 24 weeks after treatment in a modest proportion of patients [54]. VIR-2218 produced dose-dependent falls in HBsAg reaching a mean of 1.65 log10 IU/mL, although no patient lost HBsAg or developed anti-HBs [60]. In the B-Clear study, 24 weeks of bepirovirsen alone produced lasting loss of both HBsAg and HBV DNA in about 9% to 10% of patients, and responses were heavily concentrated in those who started with a low HBsAg level [61].

Two limitations need to be kept separated because they are often run together. The first is that silencing viral transcripts is not the same as removing cccDNA. RNA-based drugs switch off gene expression pharmacologically, while the cccDNA template remains intact and able to transcribe RNA, so HBsAg returns once the medication is discontinued, unless immune control has been restored during the period when antigen was low. This is why what matters for this class is how durable the response is after treatment ends rather than how low HBsAg falls during it. The second limitation is that transcripts produced from integrated HBV DNA often lack the 3′ sequences that siRNAs designed against cccDNA-derived transcripts which are targeted, so HBsAg from integrated DNA is not knocked down. HBeAg-positive patients had large decreases in HBsAg, because at that stage, most of their surface antigen is still being made from cccDNA, which the siRNA can reach. HBeAg-negative patients and chimpanzees had much smaller reductions because, by then, a large share of their HBsAg is being made from integrated DNA instead, and the siRNA cannot target that DNA. Sequencing of the integrated fragments confirmed the explanation, since many of them lacked the siRNA target sites [11,62]. Drugs aimed at conserved regions shared by all HBV transcripts, such as the S region targeted by xalnesiran, partly get around this issue, but the general point still stands. A drug that targets viral transcripts cannot reduce HBsAg below the limit set by the antigen coming from integrated DNA, which it does not recognize. Lasting HBsAg loss therefore needs either combination with something that restores immune control or an approach directed at the integrated DNA itself.

#### 5.2.4. Nucleic Acid Polymers

Nucleic acid polymers (NAPs) are phosphorothioated oligonucleotides whose antiviral activity comes from their physical and chemical properties. They are thought to interfere with the assembly and release of subviral particles. These are the non-infectious spherical and filamentous HBsAg particles that circulate in a 1000-fold to 100,000-fold excess over whole virions and are regarded as a major cause of immune exhaustion in humans. Blocking their release traps HBsAg inside the cell and causes a rapid fall in HBsAg in the blood. The idea behind the treatment is that removing circulating antigen allows HBV-specific immunity to recover. In the REP 401 study, REP 2139 or REP 2165 combined with tenofovir disoproxil and pegylated interferon produced loss of HBsAg in a substantial proportion of patients during treatment, and functional cure was maintained in a minority at 48 weeks of follow-up [63].

Two features of this class need to be discussed. The first is the ALT flares that typically occur during NAP treatment. These are due to drug toxicity. They occur at the same time as HBsAg falls, they usually normalize on their own, and they are interpreted as the return of antiviral immune activity once the antigen load drops. That interpretation is supported by the fact that they are linked to later HBsAg loss and that most reported cases were not accompanied by liver decompensation. The same pattern is seen in the immune-driven flares that precede spontaneous HBeAg or HBsAg seroconversion. Even so, flares of this size carry a real risk of decompensation in patients with cirrhosis or limited liver reserve, so they call for close biochemical monitoring and for keeping patients with advanced disease out of early-phase studies. The second feature is how weak the evidence base still is. The NAP trials have been small, largely open-label, carried out mainly at a single center in a group of Caucasian, HBeAg-negative, genotype D patients, and without a randomized comparison group receiving the same interferon and tenofovir backbone. It is therefore not possible to state with confidence that the functional cure rates were due to the NAP, since pegylated interferon and tenofovir on their own also produce HBsAg loss in a minority of such patients. The results, however, are encouraging and biologically plausible, but they should be treated as a hypothesis until larger, randomized trials in more varied populations have been performed.

#### 5.2.5. Comparing the Classes and the Case for Combination Treatment

Describing these classes one after another hides the more important point, which is that they complement each other, and that the pattern of their individual failures tells us what a curative regimen would have to contain. Table 1 sets out the comparison among all of them.

One further point applies to all of them. Each is a drug acting against a virus whose survival depends in the end on a failing immune response, and in every case where antigen has been reduced without restoring immune control, HBsAg has returned once treatment stopped. Healthy adults who achieve functional cure on their own do so through their immune system, not by suppressing replication, and this is the strongest reason to think that no purely direct-acting regimen will ever be curative. The case for combination treatment follows directly, and it is not simply a matter of adding effects together. A regimen that pairs a drug-suppressing replication, such as a nucleoside analog or a CAM, with a drug-reducing antigen load, such as an siRNA, ASO, or NAP, and with an immune-modifying drug that lets HBV-specific T-cell and B-cell responses recover attacks three separate mechanisms of persistence. Only regimens of this kind may be capable of producing responses that last after treatment ends [9,12]. REEF-1, which combined an siRNA with a CAM and a nucleoside analog, and Piranga, which combined an siRNA with an immunomodulator, are early tests of this reasoning [54,59]. Both producing functional cures in only a small minority does not mean the reasoning is wrong. It means the best partner drugs, the order and length of each part, and the patients most likely to benefit are all still unknown. Baseline HBsAg level is emerging as the most consistent predictor of response across regimens, which suggests that reducing antigen may need to come before or alongside immune treatment rather than after it [54,61].

### 5.3. Targeting cccDNA

True HBV cure requires eradication or permanent silencing of cccDNA [10]. The first-in-class orally available cccDNA inhibitor cccR08 demonstrated specific cccDNA reduction in preclinical models with sustained effects after treatment cessation. Gene-editing approaches, including CRISPR/Cas9 systems and engineered ARCUS nucleases delivered via lipid nanoparticles, have shown significant cccDNA cleavage and durable HBsAg reduction in animal models. Epigenetic silencing strategies and APOBEC-mediated cccDNA degradation via lymphotoxin-β receptor activation represent additional investigational avenues. These approaches remain in early development, and challenges related to delivery, specificity, and long-term safety must be addressed before clinical translation [10,64,65,66].

### 5.4. Immune-Based Treatment Strategies

If restoring the immune response is a necessary part of cure, then immune-based treatments may matter a great deal, and this is currently the most active area of HBV research. The immune defect in chronic hepatitis B is well described. HBV-specific CD8+ T cells are reduced in number and worn out in function, they express PD-1, CTLA-4, TIM-3, and LAG-3, and they show impaired cytokine production and abnormal mitochondria. B-cell responses to HBsAg are also defective. Long exposure to the excess of antigen carried by subviral particles is thought to drive both problems.

Immune checkpoint inhibitors try to reverse T-cell exhaustion directly. In a pilot study of patients whose virus was already suppressed, a single low dose of the anti-PD-1 antibody nivolumab, given with or without the therapeutic vaccine GS-4774, lowered HBsAg in most patients and produced lasting HBsAg loss in one, with no severe immune-related side effects [67]. This showed that the principle works, but it also exposed its limits. Checkpoint blockade can only revive T cells that still exist, and in long-standing infections, much of the HBV-specific pool has been depleted rather than merely switched off. There is also the risk of immune-mediated hepatitis in an organ that is itself the site of infection, so the margin for safety in patients with fibrosis is narrow. Checkpoint inhibition is therefore most likely to be useful as a short course given after antigen has been reduced, rather than as a treatment on its own.

Therapeutic vaccination aims to create new HBV-specific responses rather than to release existing ones. Successive versions have included protein-based vaccines, viral vectors carrying core and polymerase antigens, and heterologous prime-boost regimens that combine the two. Recombinant protein products designed to produce anti-HBs are also in development. Earlier candidates were given while circulating antigen was still high and gave disappointing results, which fits the expectation that excess antigen will tolerize the immune system rather than prime it. Current programs therefore give therapeutic vaccines after or alongside siRNA treatment that has already lowered antigen, and the combination arms of trials such as Piranga are early examples of this design. Results thus far are modest, and no therapeutic vaccine has yet shown a convincing effect on functional cure rates.

Monoclonal and broadly neutralizing antibodies against HBsAg work in a different way, combining direct clearance of antigen with possible effects on the immune response. Tobevibart (VIR-3434) is an engineered antibody aimed at a conserved part of the antigenic loop of HBsAg. It neutralizes both HBV and HDV, and in humanized mice, it controlled infection and lowered circulating HBsAg [68]. Beyond neutralizing the virus, antibody–antigen complexes may improve antigen presentation and produce a vaccine-like effect, and the antibody Fc region has been engineered to strengthen this. The main limitations are that antibody treatment removes circulating antigen without impacting the source. Also, HBsAg is produced in such large excess that keeping it suppressed needs high and frequent dosing. Combining these antibodies with an siRNA, which reduces how much antigen is made in the first place, is the logical pairing and is now being tested in patients.

Cell-based treatments aim to replace the exhausted immune response instead of repairing it by giving the patient T cells built in the laboratory to recognize the virus. T cells engineered with HBV-specific T-cell receptors, or with chimeric antigen receptors against HBsAg, can recognize both cells containing cccDNA and cells carrying integrated HBV DNA. The demonstration that antigens made from integrated DNA are displayed on liver cancer cells and can be targeted by selected T cells is particularly interesting because it connects this approach to both cure and cancer treatment [69]. Feasibility has been shown in patients with HBV-related liver cancer after transplantation. The obstacles are considerable. Liver toxicity is an unavoidable consequence if a large proportion of hepatocytes are infected. The liver actively damps down immune responses, so transferred cells are switched off and die away rather than surviving long enough to kill and clear the infected hepatocytes, and the cost and complexity of manufacturing sit awkwardly with a disease concentrated in low-income and middle-income countries.

A few other approaches are worth noting briefly. Toll-like receptor agonists, particularly oral TLR7 and TLR8 agonists, aim to produce the patient’s own interferon and revive innate and adaptive responses without the whole-body toxicity of injected interferon. Used alone, they have produced modest results, and they are now being tested in combination. Avian immunoglobulin Y (IgY) antibodies raised against HBsAg in egg yolk have been suggested as a cheap form of passive immunotherapy, with neutralizing activity reported in cell culture and in animal models. The benefits lie in the cost and ease of production compared with HBIG, but the evidence is entirely preclinical, no controlled human studies exist, and it is not known whether repeated doses of an antibody from another species would provoke an immune reaction. It would be premature to call IgY a clinical candidate. Finally, work aimed at correcting the metabolic and mitochondrial problems of exhausted HBV-specific T cells, and at reducing or altering regulatory cell populations in the liver, is at an early experimental stage but addresses the immune defect more directly than any of the approaches above.

## 6. Conclusions

Hepatitis B virus (HBV) infection is a major global health challenge that causes substantial morbidity and mortality through cirrhosis, liver failure, and hepatocellular carcinoma. Although universal vaccination and perinatal prophylaxis have significantly reduced HBV transmission, still incomplete birth-dose vaccination and limited access to screening and treatment remain some important barriers to global eradication.

Current antiviral therapies, including entecavir, tenofovir disoproxil fumarate, tenofovir alafenamide, and pegylated interferon alfa, effectively suppress HBV replication and reduce the risk of cirrhosis and HCC. However, these treatments rarely achieve functional cure because HBV persists through covalently closed circular DNA (cccDNA), integrated viral DNA, and impaired host immune responses.

Therefore, the research field is moving toward therapeutic strategies that target multiple stages of the HBV life cycle and address viral persistence. Emerging approaches, including capsid assembly modulators, small interfering RNAs, nucleic acid polymers, cccDNA-directed therapies, and immune-based strategies, provide promising opportunities for achieving durable HBsAg loss. Future progress will likely depend on rational combination therapies, improved biomarkers for patient selection, and equitable access to prevention and treatment. Integrating these advances with effective vaccination, early diagnosis, and sustained clinical care will be essential to achieving functional cure and, ultimately, the global elimination of HBV.

## Figures and Tables

**Table 1:** Comparison of treatment classes for chronic hepatitis B, showing the molecular target of each class, its effect on the main barriers to cure, and its built-in limitation.

Class	Mechanism of Action	Effect on HBV DNA	Effect on HBsAg	Effect on cccDNA and Integrated DNA	Limitations
Nucleos(t)ide analogues (ETV, TDF, TAF)	Chain termination of HBV polymerase, blocking negative-strand DNA synthesis	Strong suppression (over 90%)	Very little (HBsAg loss 1–5% long term)	None on cccDNA, none on integrated DNA	Acts after transcription, so treatment must continue indefinitely
Entry inhibitors (bulevirtide)	preS1 lipopeptide that blocks the NTCP receptor	Modest when HDV is not present	Very little	Prevents new cccDNA forming, no effect on cccDNA already present	Cannot affect cells that are already infected, relapse after stopping
Capsid assembly modulators (bersacapavir and others)	Allosteric change to core protein assembly, giving abnormal or empty capsids	Strong suppression of HBV DNA and HBV RNA	Very little	Blocks topping up of cccDNA but does not reduce the existing pool	No effect on transcription from cccDNA or integrated DNA
siRNA and ASO (xalnesiran, JNJ-73763989, VIR-2218, bepirovirsen)	RISC-mediated or RNase H-mediated breakdown of HBV transcripts	Reduction	Largest falls of any class (up to about 1.7 log10), HBsAg loss about 10% with ASO	Silences transcripts but does not remove the template	Silencing is reversible, and antigen from integrated DNA partly escapes
Nucleic acid polymers (REP 2139, REP 2165)	Amphipathic oligonucleotides that block assembly and release of subviral particles	Reduction when given with an NA	Rapid and marked fall	No direct effect	Small, largely open-label, single-centre evidence, ALT flares need monitoring
Pegylated interferon alfa	ISG induction, epigenetic silencing of cccDNA transcription, APOBEC-driven cccDNA breakdown, immune activation	Moderate suppression	HBsAg loss 3–12%	Transcriptional silencing and partial breakdown	Poorly tolerated, unsafe in decompensated cirrhosis and pregnancy
cccDNA-directed and gene editing (ccc-R08, CRISPR/Cas9, ARCUS)	Direct inhibition, cutting or epigenetic silencing of cccDNA and integrated DNA	Preclinical	Preclinical	The only class aimed at the reservoir itself	Preclinical, problems of delivery and off-target breaks, no clinical assay for liver cccDNA
Immune-based treatment (checkpoint inhibitors, therapeutic vaccines, bNAbs, engineered T cells)	Reversing T-cell exhaustion, priming new responses, neutralising antigen, transferring effector cells	Indirect	Variable, lasting loss in isolated cases	Indirect, through clearing infected cells	Much of the T-cell repertoire is deleted rather than exhausted, risk of immune hepatitis

## Data Availability

No new data were created or analyzed in this study. Data sharing is not applicable to this article.
